# Influence of three lighting regimes during ten weeks growth phase on laying performance, plasma levels- and tissue specific gene expression- of reproductive hormones in Pengxian yellow pullets

**DOI:** 10.1371/journal.pone.0177358

**Published:** 2017-05-11

**Authors:** Shunshun Han, Yan Wang, Lingyan Liu, Diyan Li, Zihao Liu, Xiaoxu Shen, Hengyong Xu, Xiaoling Zhao, Qing Zhu, Huadong Yin

**Affiliations:** 1 Farm Animal Genetic Resources Exploration and Innovation Key Laboratory of Sichuan Province, Sichuan Agricultural University, Chengdu, Sichuan, China; 2 The Statistics Bureau of Zhongjiang County, Deyang, Sichuan, China; International Nutrition Inc, UNITED STATES

## Abstract

The study was conducted to optimize lighting schedule for pre-pubertal (12 to 22 weeks) Chinese native breed Pengxian yellow pullet. A total of 414 healthy pullets (10 weeks), with similar body weight were randomly distributed into three groups (n = 138) and housed in individual cages for up to 12 weeks of age in light controlled rooms and provided normal lighting schedule (10L:14D). At 12 to 18 weeks of age, pullets were housed in three rooms, having varying lighting schedule viz. G1 (8L: 16D), G2 (10L:14D), or G3 (12L:12D). From 19th week onwards lighting schedule was gradually increased every week in incremental manner till all groups started receiving 16L:8D lighting schedule. The age at first egg, weight of first egg laid, percent peak hen day egg production, concentration of plasma luteinizing and follicle-stimulating hormones and expression of genes regulating synthesis or/and secretion of hypothalamic gonadotropin-releasing hormone-I (GnRH-I), and pituitary LH-β and FSH-β were studied during experimental period (12 to 43 weeks of age) of this study. The result indicated that pullets of long day length (G3) group had higher plasma levels of FSH and LH and also better mRNA expression that regulates synthesis or/and secretion of GnRH-I, FSH-β, and LH-β before egg laying. The age at first egg (151.3 days) in pullets of G3 group receiving longer lighting hours (12L:12D) was 8.8 days less (*P*<0.05) compared to pullets of G1 group, while it was 6.9 days less (*P*>0.05) compared to G2. However, significantly higher (*P*<0.05) plasma levels of LH and FSH in pullets of G1 as compared to pullets belonging to G3 group corresponded with the higher (*P*<0.05) cumulative egg production during the experimental period, while these attributes in G2 group didn’t differ from either G1 or G3 groups. Pullets of G1 group had significantly higher levels (*P*<0.05) of GnRH-I, FSH-β, and LH-β mRNA abundances at 43 weeks of age than other two groups and this corresponded with the percent (hen day) peak egg production (75.38%) in pullets in this G1 group that was attained at 32 weeks of age, while the peak production of 71.24% was attained at 30 weeks of age in G3 group. There was no effect of lighting schedule on body weight of pullets, recorded during experimental period, at all occasions; belonging to three groups (G1,G2 and G3) and receiving varying hours of photo-stimulation (*P*>0.05). It was inferred that the optimum lighting schedule for Chinese native breed Pengxian yellow pullets during 10 weeks of pre-pubertal growth period is short hours of photo-stimulation (i.e 8L:16D).

## Introduction

The age at which pullets sexually mature has a direct influence on their laying performance, and genetic stocks have optimal ages at which they reach sexual maturity to produce the maximum possible egg mass [1]. Among the numerous factors that can affect the time of sexual maturation, the light program (day length and light intensity) to which a flock of laying hens is subjected during the growth and production phase seems to play a critical role [2]. Pullets grown in light-proof houses are usually not held on constant day lengths during rearing, but are given some pattern of photoperiods, and most lighting programs have recommended combinations of decreasing and increasing photoperiods during different stages of the pullet’s life [3]. Koelkebeck reported that if leghorn pullets and broiler breeder pullets are grown under an increasing daylength, then sexual maturity will be enhanced which can cause egg production and blowout problems in the layer house. If pullets are grown under a decreasing daylength, then sexual maturity will be delayed [4]. It is common to rear egg-type pullets on 8-h day length until they reach 18 to 20 weeks, followed by initial weekly increments of 30 min or 1 h thereafter to a peak of 14- to 16-h day length, depending on genetics [5]. However, managers should continually evaluate the optimal light stimulation based on age for pullets, even if they are using the same strain, because there is constant selection by breeders.

When the chicken perceives day length to be sufficient to initiate reproductive development (effective minimum of 11 to 12 h), light energy is converted into nerve impulses in the hypothalamus, where it is translated into hormonal signals [6]. The extra-retinal photoreceptor and biological clock provide inputs to the final component of the hypothalamic pathways for transduction of photoperiodic information: the neurons synthesize and secrete gonadotropin-releasing hormone (GnRH), which then stimulates luteinizing hormone (LH) and follicle stimulating hormone (FSH) secretion from the pituitary gland [7]. Until now, only three types of GnRH have been found in avian brain. cGnRH-I is thought to be the main hypophysiotropic factor in the control of gonadotropin (GTH) synthesis and secretion, whereas cGnRH-II may act as neurotransmitter and cGnRH-III as a potential mediator to regulate GTH secretion [8]. The gonadotropins, FSH and LH are heterodimers that share a common α subunit (GPα) and possess hormone-specific β subunits (FSH-β and LH-β) [9]. FSH and LH are known to be key hormones in the control of reproduction and stimulate gonadal steroid production and/or secretion [10]. More specifically, LH regulates sex steroid production and ovulation, whereas FSH promotes spermatogenesis and ovarian follicle maturation in chickens and other vertebrates [11].

Pengxian yellow chickens are a unique native chicken breed from Chengdu Plain, Sichuan Province, China. This chicken breed is widely adaptable and has nutritious meat, but reproductive performances need to be improved. Therefore, to create an optimal light program for the late growth period of Pengxian yellow chickens, the effects of different light programs from 12 to 18 weeks on egg production traits and plasma FSH and LH concentrations, and gene expression of hypothalamic GnRH-I, pituitary LH-β, and FSH-β were determined in the present study.

## Materials and methods

All procedures were approved by the Animal Care and Welfare Committee of Sichuan Agricultural University.

### Chicken population

Four hundred and fourteen healthy Pengxian yellow chicken pullets of similar body weight (1072.2±58.6 g) at 10 weeks of age were divided into three light-controlled rooms (n = 138) and reared in individual cages. Birds were subjected to a feeding program with daily administration of feed and water, as recommended by the Pengxian Yellow Chicken Breeder Management Guidance.

### Light treatments

**Fig 1:**
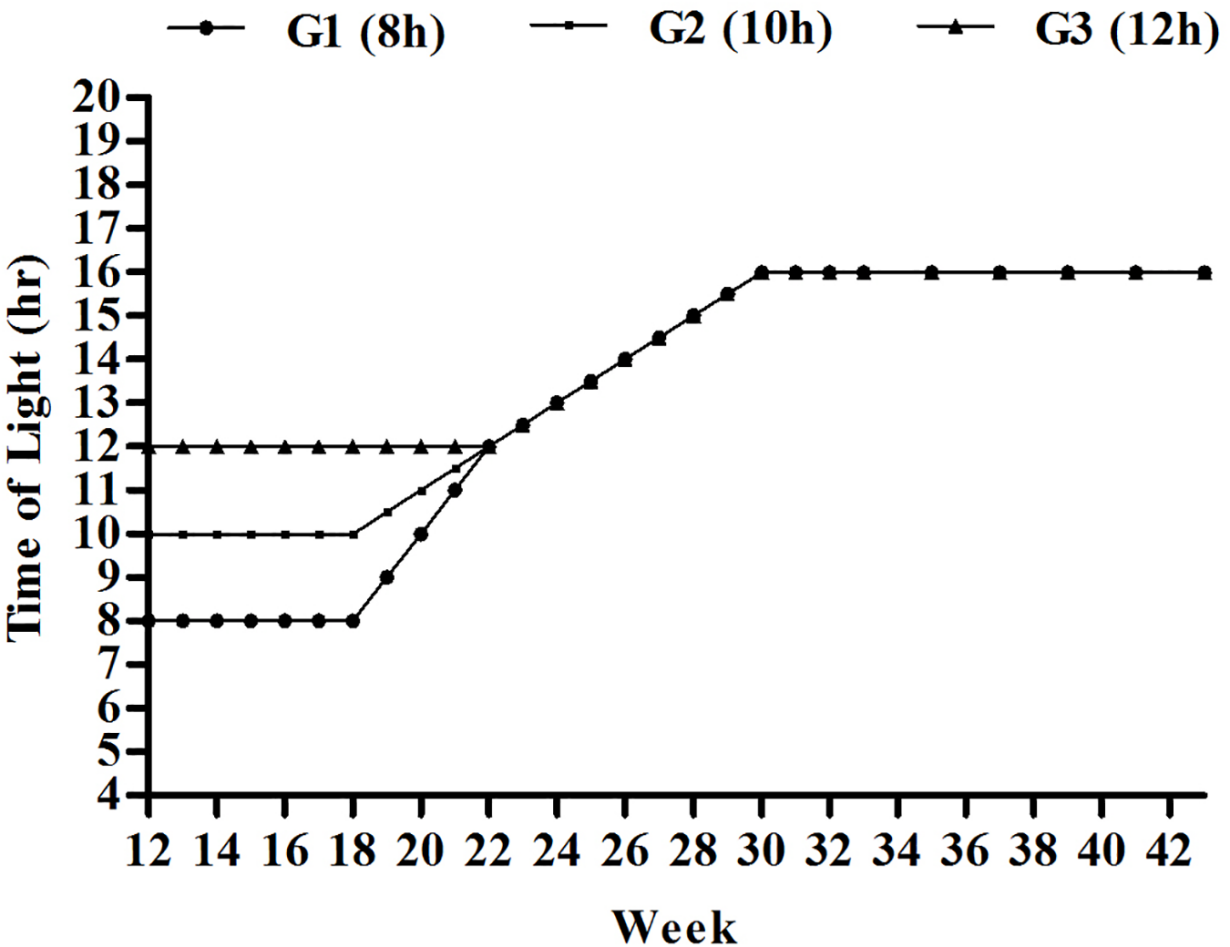
Light programs for three different treatments. From 12 to 18 week of age, 8L:16D was used in room 1 (G1), 10L:14D in room 2 (G2), and 12L:12D in room 3 (G3). From 19 to 22 week, the light programs were 9, 10, 11, 12 L in G1; 10.5, 11, 11.5, 12L in G2; and 12, 12, 12, 12 L in G3. The day length was gradually increased by 0.5 h/week from 22 week until 16L: 8D was applied in all groups.

Birds were kept under the normal photoperiod of 10 h in light (L):14 h in darkness (D) (10L: 14D) with a full light spectrum achieved by warm white fluorescent lamps (white light: 15 lux) from 10 to 12 weeks of age. Light intensities were determined by a light intensity meter (AR823; SMART SENSOR, Shenzhen, Guangdong, China). From 12 to 18 weeks of age, pullets were placed in rooms provided with different light programs: 8L: 16D in room 1 (G1, short day length), 10L:14D in room 2 (G2, medium day length), and 12L:12D in room 3 (G3, long day length). From 19 to 22 weeks, the light programs were 9, 10, 11, 12 L in G1; 10.5, 11, 11.5, 12L in G2; and 12, 12, 12, 12 L in G3. Beginning at 22 weeks, the day length was gradually increased by 0.5 h/week, until 16L: 8D was applied (Fig 1).

### Trait measurements

The age at first egg (AFE), first egg weight (FEW) and total egg production during 43 week (TE43W) was individually recorded. Thirty chickens were randomly selected to measure the fasting body weight every two weekends from 12 to 24 week, and at the age of first egg (BWFE) and the 43 week of age (BW43W).

### Hormone analysis

Heparinized blood samples were drawn from the brachial veins of the randomly selected 10 chickens at 8:00 AM every two weekends from 12 to 18 weeks of age, and every four weekends from 18 to 38 weeks, and at 43rd week. The blood was centrifuged at 3000 rpm for 15 min at 4°C. Then, the supernatant was collected to determine plasma FSH and LH concentrations. Plasma FSH and LH concentrations were measured using the commercially available ELISA kits, CSB-E06867h for FSH, and CSB-E12690h for LH (CUSABIO, Wuhan, China), according to manufacturer’s instructions for respective kits. Standards and samples were assayed in a volume of 50 μl in duplicate. The minimum detectable concentration of FSH and LH using these kits is typically less than 1 mIU/ml and 0.5 mIU/ml, respectively.

### Tissue sampling and RNA expression

Five chickens were randomly selected from each treatment and killed by cervical dislocation at the end of 18 weeks, 22 weeks (approximately 5% egg laying reached in the earliest group), 24 weeks (approximately 5% egg laying reached in the latest group), or 43 weeks. The entire hypothalamus and pituitary tissues were collected from each sacrificed chicken. All tissues were excised and rinsed in PBS, snap frozen in cryogenic tubes in liquid nitrogen, and then stored at −80°C.

Frozen tissues were pulverized in liquid nitrogen, and approximately 50 mg of powder was transferred to an RNase-free tube with TRIzol reagent and total RNA was isolated following the manufacturer’s instructions (Invitrogen, Carlsbad, CA, USA). RNA integrity, quality, and quantity analyses were performed with the Bioanalyzer 2100 (Agilent Technologies, Santa Clara, CA, USA) according to The RNA 6000 Nano chip assay was performed according to manufacturer’s instructions. All total RNA samples were stored at −80°C. Reverse transcription was performed with 2 μg of total RNA using PrimeScript RT Master Mix Perfect Real Time reaction kit (TaKaRa Biotechnology Co., Ltd. Dalian, China) according to the manufacturer’s instructions.

Real-time Polymerase chain reaction (PCR) primers were designed by Primer Premier 5 (Table 1), and the mRNA abundance of each gene was determined using a Bio-Rad CFX96 Touch real-time PCR detection system (Bio-Rad, Hercules, CA, USA). Real-time PCRs were performed in triplicate in a 10-μL volume that contained 1 μL of cDNA, 0.3 μL of reverse and forward primers (10 μM) for each gene, 3.4 μL of double-distilled H2O, and 5 μL of SsoFast EvaGreen Supermix (Bio-Rad, Hercules, CA, USA). PCR products were separated by electrophoresis on 2% agarose gel, and stained with ethidium bromide. The gene expression relative to the endogenous control (β-Actin) for each sample was calculated using the comparative 2^−ΔΔCT^ method [12].

**Table 1:** Primers used for quantitative real-time PCR of reproductive hormones genes.

Gene	Primer (forward)	Primer (reverse)	Product length (bp)	NCBI accession number
GnRH-I	ATCTGCTTGGCTCAACACTG	ATCAGGCTTGCCATGGTTTC	191	NM_001080877.1
FSH-β	GCCATCCTACTGCTCCTTCA	GCTTGGCAGTTTCTCGGTAC	154	NM_204257.1
LH-β	CCCAAAGTCATCCTACCCGT	TATGGGGCAATCTATGGGGC	169	S70834
β-Actin	:GAGAAATTGTGCGTGACATCA	CCTGAACCTCTCATTGCCA	152	NM_205518.1

### Statistical analysis

All statistical analyses were performed using SPSS 17.0 (SPSS Inc., Chicago, IL, USA). Differences between means were evaluated by Tukey’s test for multiple comparisons. Data are presented as least squares means ± standard error of the mean (SEM), and values were considered statistically different at *P*<0.05.

## Results

### Egg production and body weight

**Fig 2:**
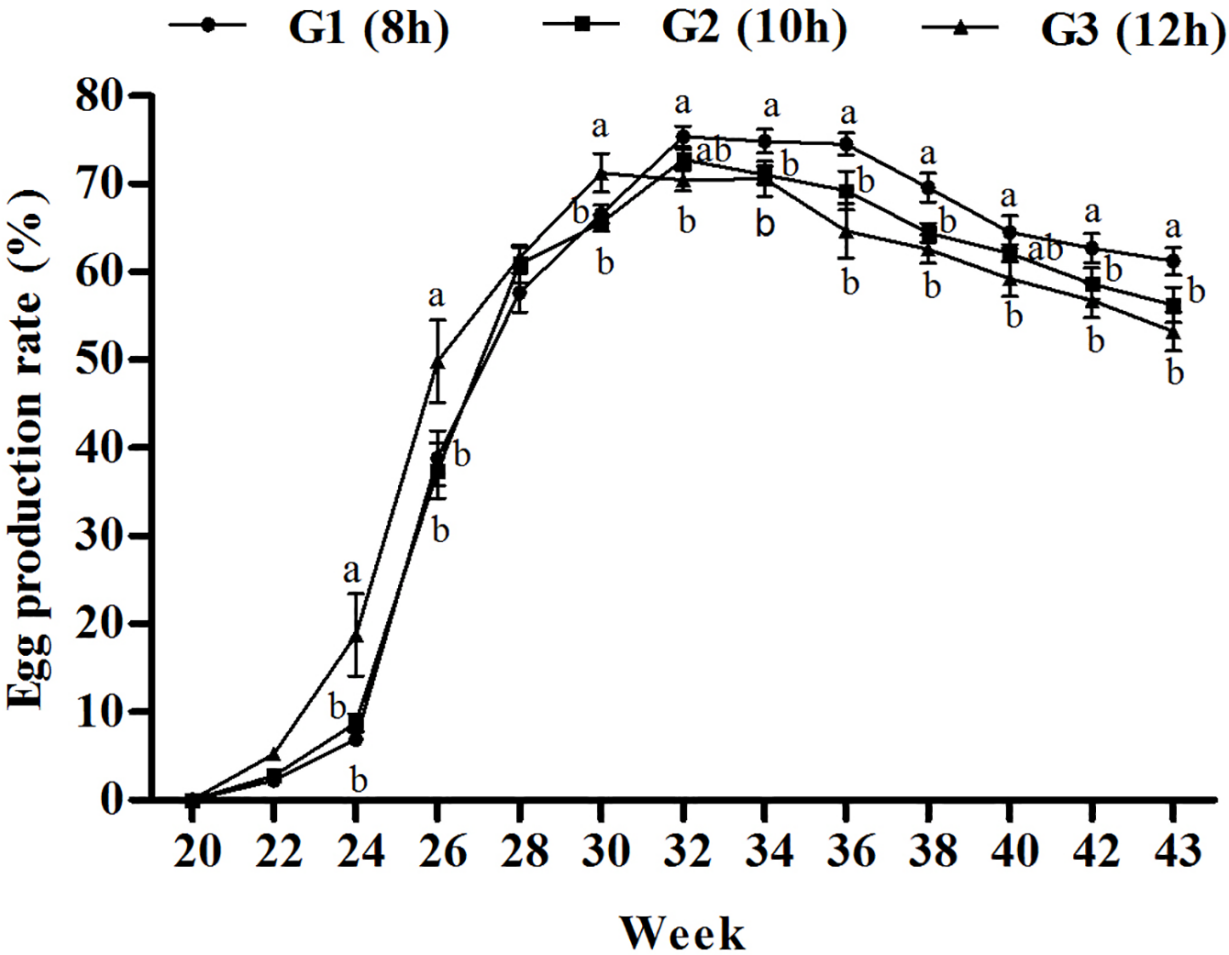
Effect of three lighting regimes during growth phase on egg production rate (%) of Pengxian yellow chicken. Lighting Regime (hours) (Light: Dark): G1- 8L: 16D (short day length); G2-10L: 14D (medium day length (10 h); G3-12L: 12D (long day length). Data are presented as mean ± standard error of the mean (*n* = 10). Values with different letters are significantly different (*P*<0.05).

Laying performances are presented in Fig 2 and Table 2. Laying performance was significantly affected by lighting program. The average age at first egg (AFE) in the long lighting photo-stimulated group (G3) was 8.8 days lower (*P*<0.05) than short G1 program, while it was 6.9 days lower (*P*>0.05) than G2 program, but the first egg weight (FEW) was lower (*P*<0.05) compared with the other two light-treatment groups. Egg production rate rapidly increased in G3, and peaked at 30 weeks, which was 2 weeks earlier than other two treatments (32 weeks), but the rate (71.22%) at peak was lower than that in G1 (75.38%) and G2 (72.77%). Cumulative egg production during the 43 weeks of the experimental period was greater in the short light photo-stimulated group (G1; 98.7 ± 2.6) than in the medium (G3; 91.2 ± 3.7) and long (G3; 93.4 ± 3.4) light photo-stimulated groups.

**Table 2:** Laying performance of Pengxian yellow chicken exposed to three lighting regimes during their growth phase.

Lighting regime	AFE (d)	BWFE (g)	FEW (g)	TE43W	BW43W (g)
G1(8 h)	163.2±3.2^a^	2292±36.8	40.5±0.6^a^	98.7±2.6^a^	2763±6.3
G2(10h)	161.3±4.5^ab^	2238±39.3	40.2±0.9^a^	93.4±3.4^ab^	2690±7.6
G3(12h)	154.4±4.7^b^	2209±41.8	38.0±1.0^b^	91.2±3.7^b^	2637±8.1

AFE = age at first egg; BWFE = body weight at first egg; FEW = first egg weight; TE43W = total egg production during 43 weeks; BW43W = body weight at 43 week.

Lighting Regime (hours) (Light: Dark): G1- 8L: 16D (short day length); G2-10L: 14D (medium day length (10 h); G3-12L: 12D (long day length)

Data are presented as mean ± standard error of the mean (*n* = 123).

Values with different superscript letters differ significantly (*P*<0.05).

However, there was no effect on body weight from 12 to 24 weeks, and at AFE and 43 weeks among the three groups after photo-stimulation (*P*>0.05; data not shown).

### Plasma FSH and LH concentrations

**Fig 3:**
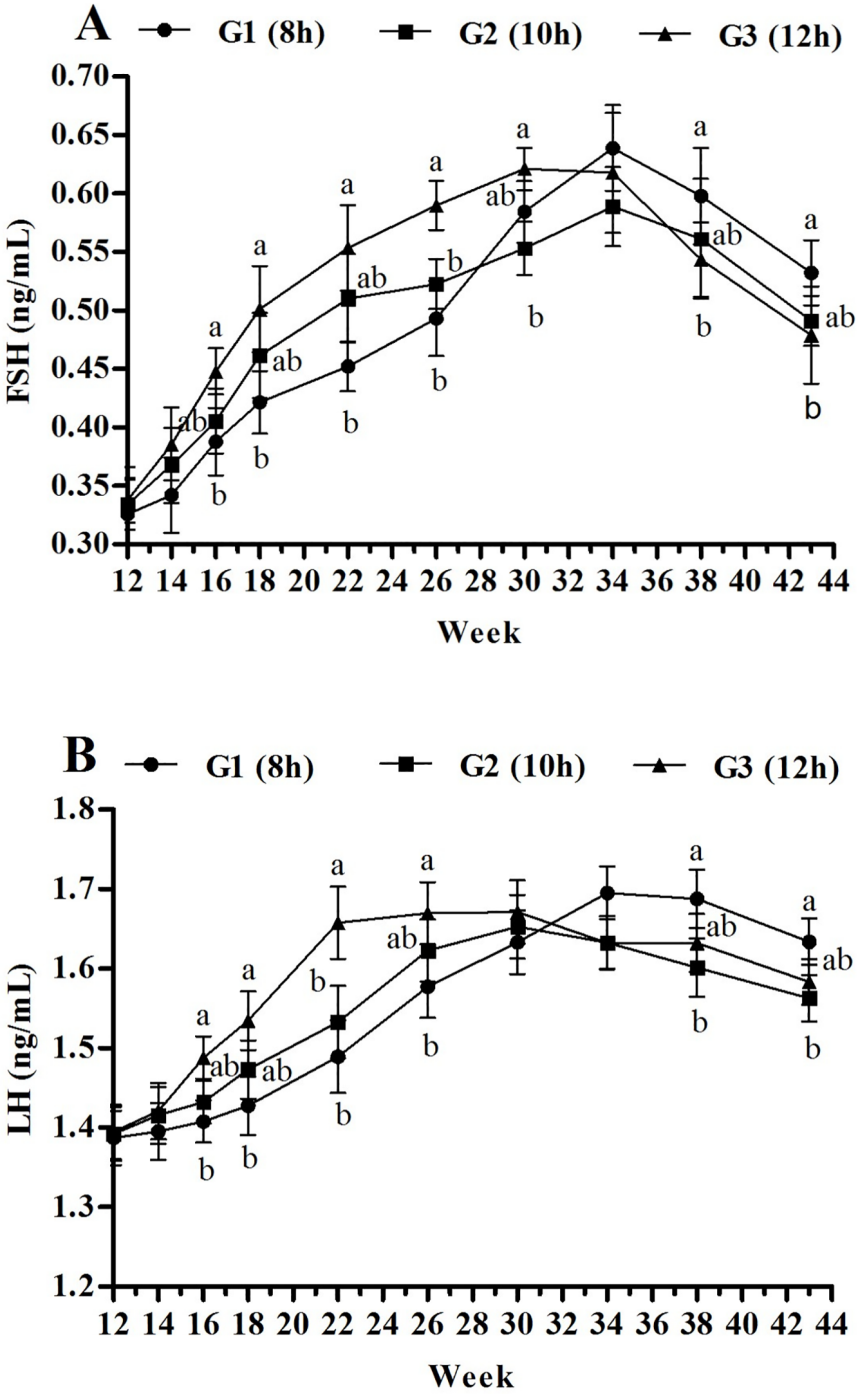
**Effect of three lighting regimes during growth phase on plasma FSH (A) and LH (B) concentration of Pengxian yellow chicken.** Lighting Regime (hours) (Light: Dark): G1- 8L: 16D (short day length); G2-10L: 14D (medium day length (10 h); G3-12L: 12D (long day length). Data are presented as mean ± standard error of the mean (*n* = 10). Values with different letters are significantly different (*P*<0.05).

The plasma FSH and LH concentrations first increased and then decreased in all treatment groups (Fig 3). The plasma FSH and LH concentrations were the same in all three groups at 12 and 14 weeks. However, the lighting programs began to have an impact on plasma FSH and LH concentrations from 16 weeks.

Plasma FSH concentrations were higher in the long lighting (G3) than short lighting (G1) photo-stimulated group from 16 to 30 weeks (*P*<0.05; Fig 3A). Then, the FSH concentrations were similar among the three groups at 34 weeks, but G1 had higher FSH concentrations at 38 and 43 weeks (*P*<0.05).

Plasma LH concentrations were higher in G3 from 16 to 26 weeks (*P*<0.05), whereas the reversed results were found from 38 to 43 weeks, when G1 had higher LH concentrations (*P*<0.05; Fig 3B). Otherwise, there were no differences found among the treatments at 30 and 34 weeks.

### Gene expression studies

**Fig 4:**
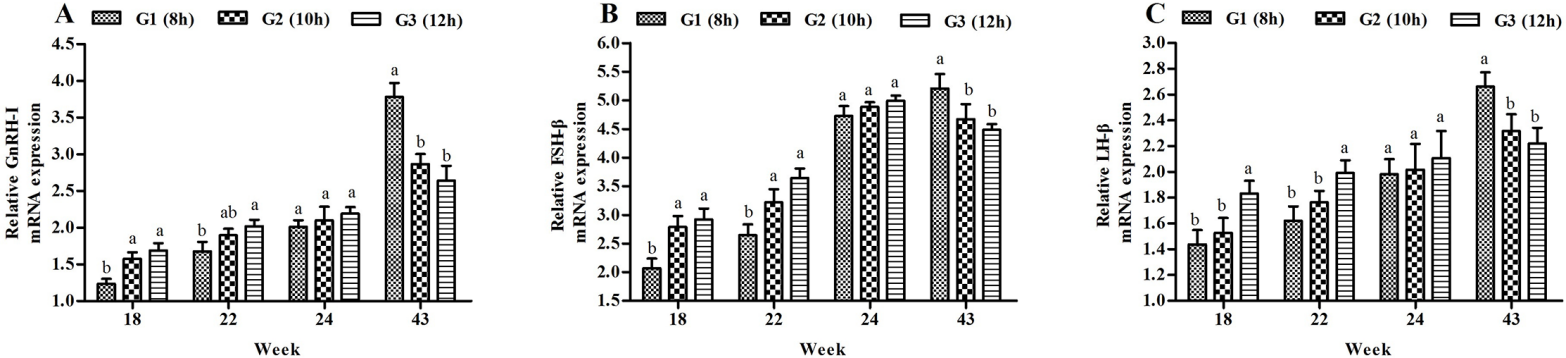
**Effect of three lighting regimes during growth phase on hypothalamic GnRH-I (A), and pituitary LH-β (B) and FSH-β (C) mRNA expression in Pengxian yellow chicken.** Lighting Regime (hours) (Light: Dark): G1- 8L: 16D (short day length); G2-10L: 14D (medium day length (10 h); G3-12L: 12D (long day length). Data are presented as mean ± standard error of the mean (*n* = 5). Values with different letters are significantly different (*P*<0.05).

Photo-stimulation significantly impacted the expression of hypothalamic GnRH-I, and pituitary LH-β and FSH-β transcripts (Fig 4). At 18 weeks of age, GnRH-I and FSH-β mRNA expression was higher (*P*<0.05) in medium (G2) and long lighting (G3) photo-stimulated groups than in the short lighting treatment group (G1), and the G3 had higher (*P*<0.05) LH-β mRNA abundance compared with the other two groups. At the age of 22 weeks, the three genes had entirely different expression patterns. The mRNA expression of GnRH-I in G3 was higher than that in G1 (*P*<0.05), but similar to that of G2. G2 and G3 had the same FSH-β mRNA expression, which was higher than that in G1 (*P*<0.05). The LH-β mRNA abundance was similar in G1 and G2, but lower than that in G3 (*P*<0.05).

The mRNA expression patterns of GnRH-I, FSH-β, and LH-β were similar in 24 and 43 weeks. There were no significant differences found among three groups in any of the genes at 24 weeks. However, expression of the three genes in G1 were higher (*P*<0.05) than those in G2 and G3 at 43 weeks.

## Discussion

Seasonal reproduction of birds is the adaptation to achieve maximal survival, regulated by the endogenous circannual rhythm, which is synchronized by periodically changing environmental factors, of which photoperiod is thought to be the most important [13]. In domestic fowl, the seasonality of reproduction has been eliminated by artificial lighting programs, which not only can induce out-of-season lay to balance production over the year, but can maximize total number of eggs laid during a certain period of time [14]. The lighting program to which a flock of breeders is subjected during the growth and production phase is a key factor in determining the onset of sexual maturity and egg production, especially during the growth period. Lighting programs (photoperiod, light intensity, wavelength etc.) for pullets kept in confined poultry houses must be designed so as to guarantee optimal growth and efficient preparation for the laying period, largely independent of the season [15]. In this study, we designed three different lighting programs during the late growth period (12 to 18 weeks) in Pengxian yellow chickens, and found 8L: 16D was the optimal photoperiod, which was consistent with–earlier observations that a constant 8 hours of light and 16 hours of dark (8L:16D) during the rearing period (0–18 weeks) was the most convenient lighting programme for the egg-type strains [16].

Light is a major determinant of the timing of sexual maturity in domestic pullets. The age at first egg and laying peak advanced with increased day length during the growth phase in our study; these findings are consistent with those of Lewis et al. (1999), who found that transferring pullets from short to long days tends to stimulate ovarian activity, whereas transferring from long to short days suppresses ovarian development [17]. Additionally, our results are in accordance with those of Lewis et al (1996), who found that age at first egg can be advanced by approximately 5 weeks if photoperiod is increased from 8 to 13 h at 63 days of age compared with a constant short day length [18]. Our findings are also similar to those of Chen et al (2007), who found that photoperiod had an effect on the timing of sexual maturity, and the AFE was 5.7 days earlier for hens exposed to the 17L:7D photoperiod than those exposed to an 11L:13D photoperiod at 20 weeks [5].

However, the cumulative egg production during our 43 weeks experimental period was lowest in G3, even though the AFE was the earliest. Not surprisingly, because overall egg size can be manipulated with a lighting program during the rearing period, and advanced sexual maturity would reduce egg mass throughout the laying period because of aging of the reproductive tract and reduction in the rate of recruitment or yellow-yolky follicles as well as an increased incidence of follicular atresia, internal ovulation and the production of membranous or soft shelled eggs [19,20]. Additionally, egg weight (EW) was lightest in the earliest laying group, which supports the idea that that EW is closely related to AFE [21].

In our study, no significant differences were observed among the three treatments based on the average live body weights of chickens during the growth period and 43 weeks, which was consistent with the results of Banks and Koen (1998), who did not find a significant effect of lighting system during the growth phase on the average live body weight of birds during the production stage [22]. However, our results regarding the effect of photoperiod on body weight were not consistent with substantial scientific evidence that longer day length during the growth period results in higher feed intakes and heavier body weights, because appetite was stimulated by photoperiod, which led to rapid early growth and enhanced skeletal development [17,23]. The variation in our results may be because a day length of 8 h provided enough light for normal feed and water access, which meets the requirement for growth development, and the longer daylength possibly may not increase the feed intake in our pullets. Additionally, there is a strong negative relationship between body weight and reproductive efficiency, therefore, the body weight of growth chickens should be controlled within reason.

Photoperiodic responses are dependent on interactions between endogenous circadian clocks and encephalic photoreceptors [24]. Unlike mammals, birds do not use melatonin to relay photoperiodic information; instead they obtain it directly through photoreceptors located within the medio-basal hypothalamus [25]. It is well known that the hypothalamic-pituitary gonadal (HPG) axis regulates the reproductive activities, because GnRH in the hypothalamus controls the secretion of FSH and LH, which, together with other hormones, regulate the reproductive seasonality of birds that are both long- and short-day breeders [26]. Lewis et al (2005) suggested that plasma LH and FSH concentrations during the rearing period might be useful predictors of sexual maturity and egg production rate in chickens [27]. Similarly, it was stated that the light stimulus will be initiated when light falls in the eye of the chicken, and the photon energy converts into neural impulses in the hypothalamus, then induces the release of LH and FSH hormones from the pituitary, which in turn causes increased growth of the ova [28].

We demonstrated changes in plasma FSH and LH concentrations from 18, 22, 24, and 43 weeks. All data sets showed that hormonal changes under different photoperiods were closely related to reproduction. Pullets reared in long day length displayed higher plasma FSH and LH concentrations in the initial laying stage. This concur with Sharp’s, who reported similar increase in level of these hormones when laying hens were first exposed to short photoperiods and then transferred to long photoperiods. However, when the procedure was reversed, the FSH and LH concentrations decreased [29]. Similar differences were found in FSH-β and LH-β mRNA expression in the pituitary gland. Photo-stimulation induced FSH-β and LH-β mRNA expression before the first egg was laid. This could have occurred because selective photo-stimulation of long day length that increased GnRH secretion and GnRH-I mRNA abundance through direct connection between the hypothalamic photoreceptor and GnRH synapses [30], and led to a release of LH and FSH with increase of FSH-β and LH-β mRNA expression in the pituitary [31]. Earlier studies have concluded that photo-stimulatory inputs to GnRH neurons have the potential to increase GnRH-I mRNA transcription or stability [32], and subsequent work in quail and turkey demonstrated that GnRH-I mRNA expression was also increased by photo-stimulation [33,34]. However, the plasma FSH and LH concentrations, and FSH-β and LH-β mRNA abundance were higher in G1 during mid- to late laying, which is likely because of the self-adjustment ability in chickens when photoperiod was the same in the three groups from 22 weeks.

## Conclusion

Based on the findings of this study it was concluded that day length during the growth period (12 to 18 weeks) can affect laying performance and these effects are mediated through alteration in the concentrations of gonadal hormones, which in turn is regulated through expression of related genes in the hypothalamic-pituitary-gonadal axis of female Pengxian yellow chickens. The optimal photoperiod for enhanced egg-laying in Pengxian yellow chickens of 12 to 18 weeks was found to be 8L: 16D.
